# Myocardial Infarction With Non-obstructive Coronary Arteries (MINOCA) in a Middle-Aged Male: A Case Report

**DOI:** 10.7759/cureus.109178

**Published:** 2026-05-19

**Authors:** Chirag Lodha, Eric J Basile

**Affiliations:** 1 Internal Medicine, University of South Florida Morsani College of Medicine, Tampa, USA; 2 Cardiovascular Disease, University of South Florida Morsani College of Medicine, Tampa, USA

**Keywords:** cardiac mri, minoca, mri cardiac, stemi

## Abstract

Myocardial infarction with non-obstructive coronary arteries (MINOCA) represents an uncommon clinical presentation, particularly among male patients with significant comorbidities.

This case report describes a 64-year-old male with a complex medical history, including coronary artery disease (CAD), hypertension, chronic kidney disease stage II, heart failure with improved ejection fraction, paroxysmal atrial fibrillation, peripheral arterial disease, obstructive sleep apnea (OSA), and obesity, who presented with non-ST-elevation myocardial infarction (NSTEMI) and positive high-sensitivity troponin titers. Despite angiographic findings revealing no obstructive culprit lesion, cardiac magnetic resonance imaging (MRI) confirmed acute myocardial infarction. Because there was no acutely occluded lesion with angiographic evidence of myocardial infarction, he was diagnosed with MINOCA. Management was adjusted to include dual antiplatelet therapy (DAPT) with clopidogrel and anticoagulation, alongside guideline-directed medical therapy for heart failure. After initiation of therapy for heart failure and acute coronary syndrome (ACS), he remained stable until discharge, with plans for outpatient follow-up to monitor myocardial recovery and further management of comorbidities. This case highlights a common diagnosis in an uncommon population, underscoring the need for advanced imaging to better characterize this patient population.

## Introduction

Myocardial infarction with non-obstructive coronary arteries (MINOCA) accounts for 5% to 15% of acute myocardial infarctions and shows a well-documented female predominance, so its occurrence in males with extensive comorbidities stands out as a notable exception [1,2]. It is defined as a myocardial infarction without angiographic evidence of a culprit lesion contributing to the acute infarction, with it being confirmed by advanced imaging studies such as cardiac magnetic resonance imaging (MRI) [2]. Clinicians define MINOCA as acute myocardial infarction without obstructive coronary artery disease (CAD), meaning stenosis less than 50% on angiography [1]. This can also be caused by coronary thromboembolic events secondary to atrial fibrillation or a hypercoagulable state [3]. Multiple pathogenic mechanisms contribute, including microvascular dysfunction, plaque erosion without significant stenosis, and coronary vasospasm [4]. This patient's profile, marked by obesity, hypertension, chronic kidney disease, heart failure, atrial fibrillation, peripheral arterial disease, and sleep apnea, highlights how these factors converge to trigger MINOCA despite non-obstructive arteries [5,6]. Advanced imaging like cardiac MRI proves essential for confirming infarction patterns in such cases [7,8].

The rarity of MINOCA in a middle-aged male with such comorbidities challenges typical demographic patterns [4]. Established risk factors such as obesity drive endothelial dysfunction and accelerate atherosclerosis [3]. Hypertension and chronic kidney disease add ischemic risk through vascular remodeling, while atrial fibrillation raises thromboembolic concerns [3]. Peripheral arterial disease signals systemic atherothrombosis [5]. Cardiac MRI can often be revealing, with viable myocardium and reduced ejection fraction pointing to microvascular or embolic causes [7]. Tailored therapy addressing these elements led to clinical improvement and stabilization, emphasizing individualized approaches over one-size-fits-all strategies [4,9]. Males with heart failure and atrial fibrillation face amplified risks from these interactions, as atrial fibrillation leads to increased myocardial oxygen demand as well as dyssynchrony with the left ventricle, impeding forward flow [6]. This is compounded in heart failure with reduced ejection fraction, with a backup of volume into the left atrium causing myocardial stretching and arrhythmias [6]. Cellular pathways such as dysregulated autophagy and apoptosis likely play roles in myocardial injury [10]. Coronary thromboembolism emerges as a key MINOCA cause, demanding specific diagnostic focus [3]. This case shows the multifactorial nature of MINOCA, influenced by inflammation, endothelial dysregulation, and other atherosclerotic risk factors, where non-obstructive disease masks acute events that may go underrecognized [2]. There exists a gap in the literature regarding whether to pursue an individualized approach to MINOCA treatment or manage it similarly to other acute coronary syndromes (ACS), as there is no clear consensus on its management [9]. This case study aims to show an emerging diagnosis in an atypical population and its subsequent management, as well as emerging imaging and treatment modalities. 

## Case presentation

A 64-year-old male with a history of CAD with chronic total occlusion (CTO) of the right coronary artery (RCA), hypertension, chronic kidney disease stage II, heart failure with improved ejection fraction, and paroxysmal atrial fibrillation presented with chest and back pain. He stated that both started within the last few hours without any inciting factor, such as exertion; however, he said that resting helped the chest pain improve slightly. His labs were significant for a troponin of 2,100 ng/L on admission, which subsequently increased to 4,036 ng/L before peaking at 11,646 ng/L (Table 1). His vitals were significant for a blood pressure of 135/95, with a heart rate of 72 beats per minute (BPM).

**Table 1:** Patient's troponin trend on admission, 3 hours, and 6 hours.

Lab value	On admission	3 hours	6 hours	Normal value
Troponin level (ng/L)	2,100	4,036	11,646	<50

**Figure 1:**
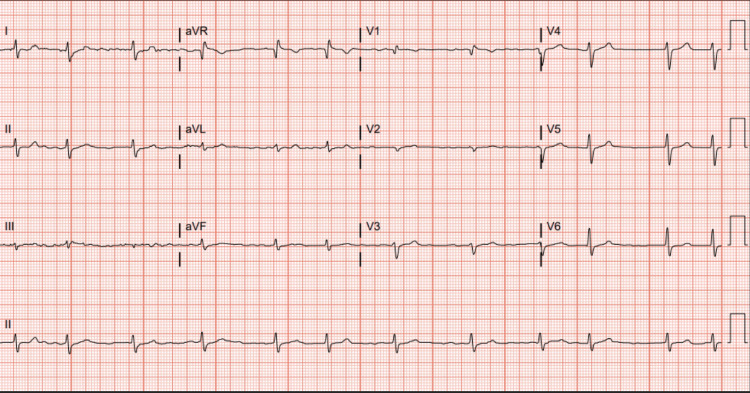
ECG showing a new developing right bundle branch block (RBBB).

His electrocardiogram (EKG) showed a developing right bundle branch block (RBBB) without signs of ST-elevation (Figure 1). Due to a new bundle branch block in the setting of chest pain and elevated troponin, it was deemed that he had an ACS at this time. He was admitted with a principal diagnosis of non-ST-elevation myocardial infarction (NSTEMI) based on an increasing troponin, transient ECG changes (Figure 1), and symptoms of chest pain radiating to the arms and back. At this time, the patient developed worsening bradycardia at 40-45 BPM with a decrease in his blood pressure to 105/79. Due to concerns of worsening ischemia contributing to his change in clinical status, the decision was made to admit him to the coronary care unit for worsening bradycardia and hypotension.

**Figure 2:**
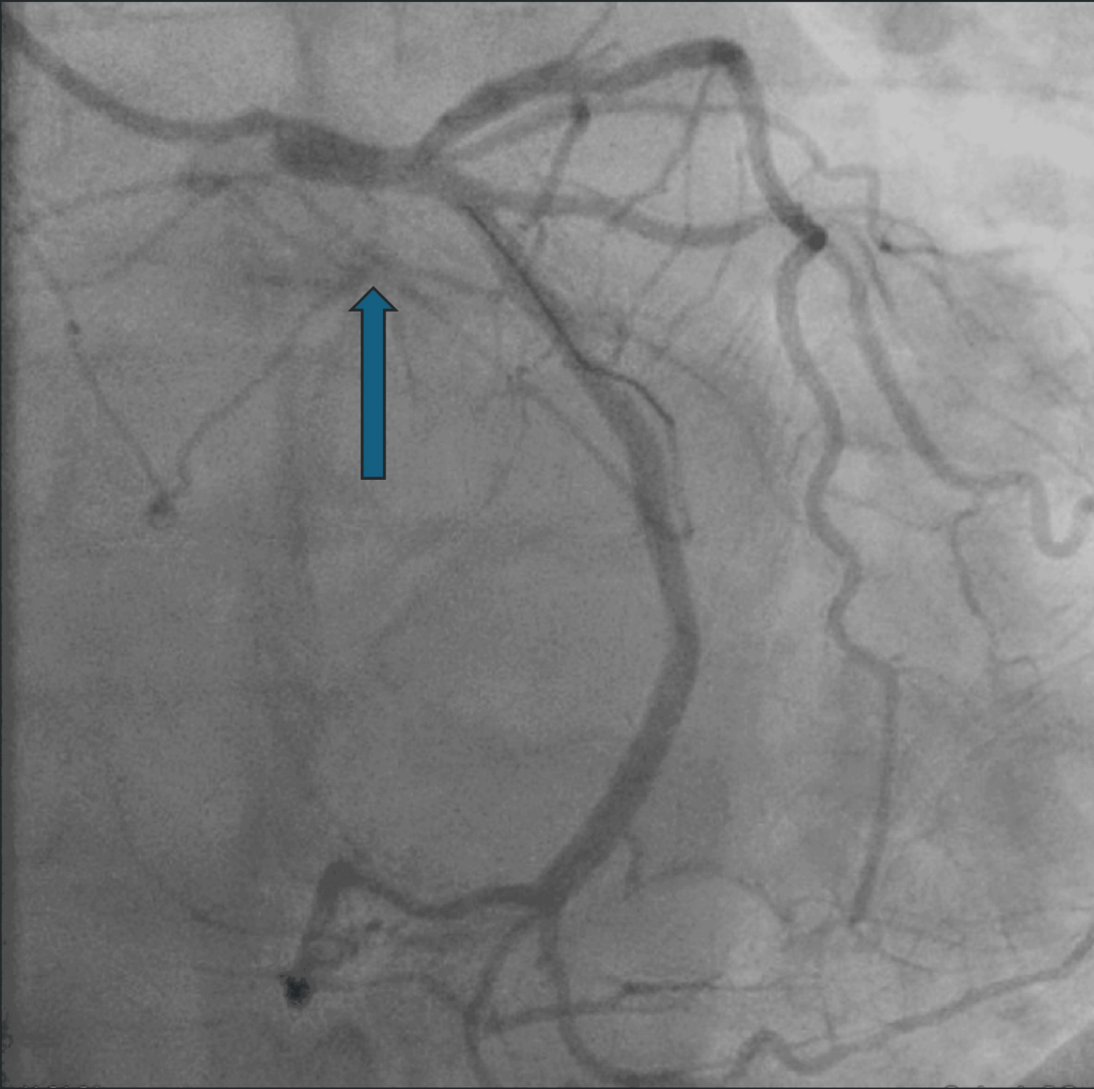
Left heart catheterization (LHC) showing left-to-right collaterals without a culprit lesion. The collaterals begin at the arrow (left-sided collateral) and continue to the right-sided circulation (left side of the screen). The arrow indicates collateral blood supply from the left-sided circulation to the right-sided circulation.

Initial management included aspirin, high-intensity statin, metoprolol, intravenous heparin, and nitroglycerin infusion. He was monitored in the cardiac intensive care unit with telemetry and serial troponins. His blood pressure improved after a 500 mL bolus of 0.9% normal saline. Due to concern for aortic dissection, given his simultaneous back pain, a computed tomography (CT) angiogram of his chest was obtained, showing no aortic dissection or pulmonary embolism. Initial transthoracic echocardiogram showed preserved left ventricular ejection fraction (LVEF) (60%-65%) without regional wall motion abnormalities and mildly to moderately reduced right ventricular (RV) function. He underwent left heart catheterization, which demonstrated mild to moderate diffuse disease in the left system, known RCA CTO with left-to-right collaterals, and no culprit lesion to explain the acute presentation (Figure 2). 

**Figure 3:**
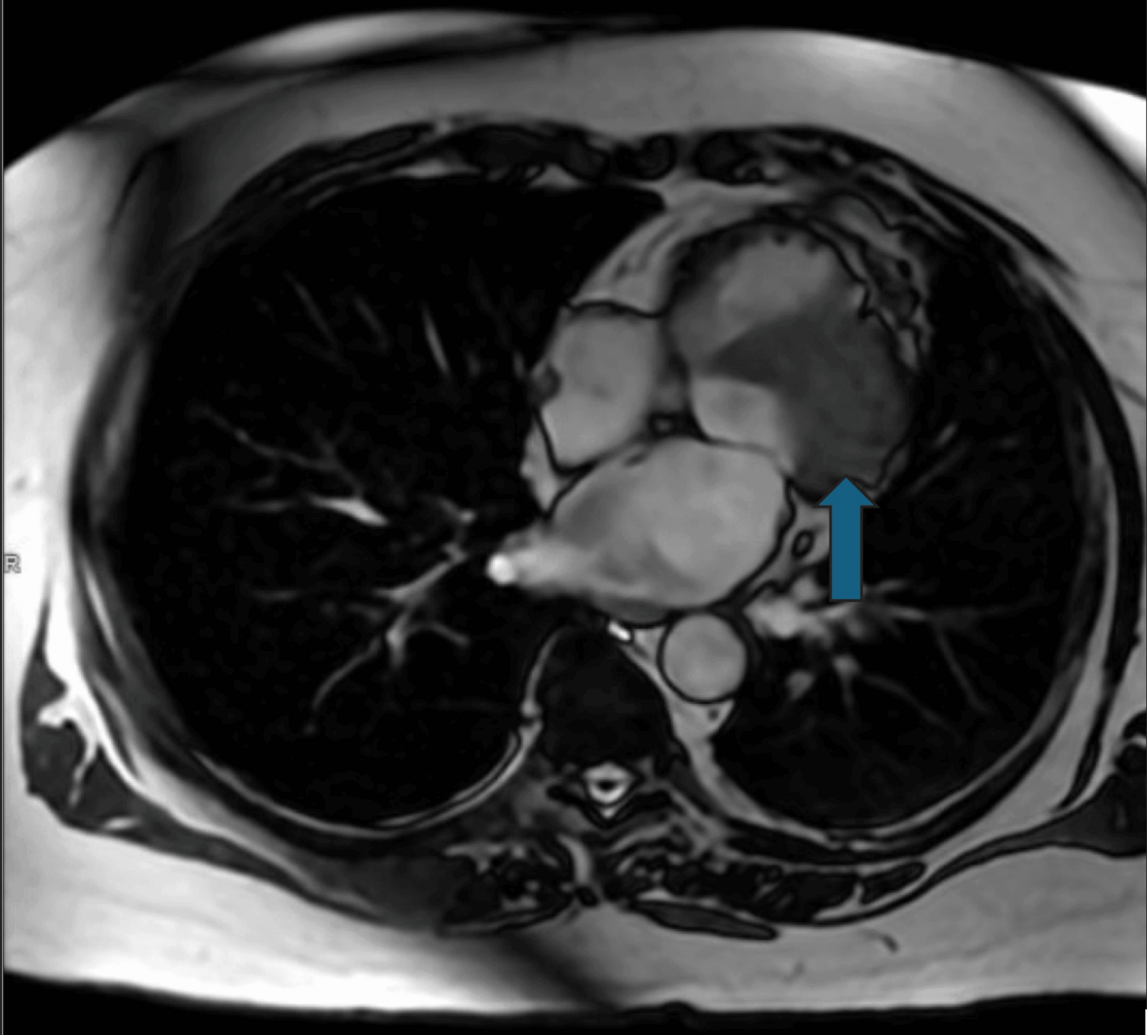
Cardiac MRI showing acute infarction. Arrow showing acute infarction.

Due to the absence of an obvious lesion causing his NSTEMI, as there was no high-grade stenosis or acute thrombus, it was thought that he had MINOCA. For this reason, he was sent for a cardiac MRI. This revealed acute myocardial infarction in the RCA/LCx vascular territory with predominantly viable myocardium, moderate global hypokinesis, septal dyskinesis, and reduced LVEF (39%), along with moderate RV dysfunction (EF 29%) (Figure 3). 

Following the cardiac MRI, aspirin was discontinued, and clopidogrel was initiated, with continuation of apixaban for atrial fibrillation and ACS. Guideline-directed medical therapy for heart failure with reduced ejection fraction was started, including switching to metoprolol succinate and adding sacubitril-valsartan, spironolactone, and empagliflozin, all of which were tolerated without adverse effects or worsening renal function. Ezetimibe was added to atorvastatin for aggressive lipid lowering.

Cardiology recommended continuation of dual therapy with clopidogrel and Eliquis for at least one year, ongoing antianginal therapy, aggressive risk factor modification, and outpatient cardiac rehabilitation and follow-up. 

## Discussion

MINOCA remains an emerging topic within cardiology, increasing in prevalence and incidence due to more sophisticated imaging modalities. Typically, this pathology is seen in younger women, which is why this case in a middle-aged male is unique in nature. Some registries suggest a prevalence of 5%-25%, but more recent studies have suggested approximately 8% [11]. MINOCA remains clinically relevant due to the prognosis of mortality being higher in terms of non-cardiac death when adjusting for one-year survival, as seen in the ACUITY trial [11]. Of note, those with epicardial vasomotor abnormalities have a higher risk of cardiovascular events, while those with angina and microvascular spasm have comparably better outcomes [11]. Therefore, regardless of demographics, if clinical suspicion remains high for MINOCA, it is imperative to risk-stratify and identify patients at higher risk [12].

The inflammatory component of MINOCA is also emerging as a leading hypothesis, characterized by oxidized lipoprotein particles and interleukins while being mediated by platelets, leading to a pro-thrombotic state and a higher risk of cardiovascular events [12]. Higher high-density lipoprotein (HDL) levels were crucial in these patients from a primary prevention standpoint [12]. Studies have shown that major adverse cardiovascular events (MACE) were higher in those with higher titers of inflammatory markers, as opposed to similar patients with lower levels [12]. Additionally, these markers were independent risk factors for MACE in patients with MINOCA, further suggesting an inflammatory hypothesis for the pathogenesis of MINOCA as opposed to hyperresponsive coronary vasculature to vasoconstrictive mediators [12].

MRI remains the gold standard for the diagnosis of MINOCA, as MRI helps delineate tissue characteristics and confirm an acute infarction when the etiology of elevated troponin remains unclear in more complex patients [13]. Although this case reports MINOCA in an atypical demographic, it is important to note that women have a greater risk of mortality after an acute myocardial infarction, as well as a higher five-year incidence of heart failure and stroke afterward [13]. Therefore, it is important to identify ACS promptly and consider advanced imaging to further delineate an uncertain cause of coronary injury [13]. Cardiac MRI can be costly to obtain, as well as not easily available at other institutions, and clinical trials have not clearly delineated a standard algorithm for the management of MINOCA, which remains an uncharted field [14]. The PROMISE trial has suggested multiple modalities to help diagnose MINOCA, such as optical coherence tomography (OCT), intracoronary acetylcholine provocation, as well as the potential for microRNA sampling [14]. Patients with MINOCA were also shown to have worse mental health and quality of life after an event compared to placebo patients, suggesting a possible affective disorder component similar to post-myocardial infarction depression and anxiety [15].

Treatment is guided by risk factor modification and medical therapy depending on the initial provoking factor causing MINOCA [9]. The PROMISE trial showed that by pursuing a stratified approach, these patients had significantly improved perceived quality of life, decreased anginal events, and better functional status [9]. However, this trial did not find a difference in mortality between this stratified approach and the standard-of-care group, which received dual antiplatelet therapy (DAPT), 81 mg aspirin, and a high-intensity statin [9]. Patients in the stratified group were prescribed DAPT if the infarction was deemed secondary to an unstable plaque, a non-dihydropyridine calcium channel blocker for vasospasm, or anticoagulation for those with a thromboembolic cause [9]. Although there was no difference in mortality, this stratified approach has shown great promise in improving symptomatic management and quality of life. Further clinical trials with larger sample sizes are necessary to determine whether an individualized treatment approach would make a significant difference in terms of MINOCA mortality.

A limitation of this case study is that it involves one patient, who represents an otherwise uncommon population in which MINOCA is seen. It is therefore difficult to make generalizations to larger patient populations based on the medical decision-making in this case report. The management was relatively vague, as there was no definite overt cause other than coronary vasospasm; however, this was managed similarly to the PROMISE trial protocol. By addressing coronary vasospasm based on the aforementioned trial, providers were optimistic that the patient would receive treatment similar to the standard of care, with an improved quality of life and fewer anginal events. 

## Conclusions

In conclusion, MINOCA is a rapidly emerging topic within cardiology, with a lack of robust clinical trials to help guide its multifaceted management. Although it remains a relatively uncommon diagnosis in males, females have significantly worse cardiovascular outcomes, with a higher incidence of heart failure and MACEs after an acute MINOCA event. The gold standard for identification is cardiac MRI, which shows an infarction pattern without any clear obstructive lesion. Treatment is guided similarly to other ACS syndromes, with an emphasis on aggressive risk factor modification with statin therapy and management of other comorbidities such as diabetes and hypertension. Emerging modalities to help identify MINOCA besides cardiac MRI show promise, including acetylcholine provocation testing as well as OCT. As trials continue to emerge, evidence points strongly toward an individualized approach to cardiac care in these patients. Further research must be done to determine whether pursuing a more individualized approach to MINOCA provides a mortality benefit rather than only a symptomatic benefit that could be attributed to placebo.
